# Racial Disparities in Cognitive Aging Among Older Black and White Men in the US

**DOI:** 10.1002/alz70860_101686

**Published:** 2025-12-23

**Authors:** Roland J. Thorpe, Boeun Kim, Keith E. Whitfield, Sarah L Szanton

**Affiliations:** ^1^ Johns Hopkins University, Baltimore, MD, USA; ^2^ University of Iowa, Iowa City, IA, USA; ^3^ University of Nevada, Las Vegas, Las Vegas, NV, USA

## Abstract

**Background:**

Cumulative exposure to social, economic, and environmental hazards throughout the life course contributes to accelerated aging in minoritized groups, potentially driving racial differences in cognitive aging. Despite this, racial differences in cognitive aging, particularly among older men are not well understood. This study examined racial disparities in baseline cognitive function and rate of change over time among older men racialized as Black or White.

**Method:**

This study utilized data from the 2011‐2021 National Health and Aging Trends Study (NHATS), a nationally representative cohort of US Medicare beneficiaries aged 65 years and older. The analysis included Black and White men living in community settings without a dementia diagnosis at baseline (577 black men and 1972 White men). Cognitive function was assessed using a composite score (range: 0–33) that incorporated orientation (assessing the date, month, year, and day of the week, as well as naming the president and vice president), executive function (evaluated through the clock‐drawing test), and immediate and delayed memory (measured using a 10‐word recall test). Higher scores indicated better cognitive performance. Race was determined based on self‐identified non‐Hispanic Black and non‐Hispanic White categories. The survey weighted multilevel mixed‐effects models were fitted to examine racial differences in cognitive function and its trajectories, adjusting for age, educational attainment, income, number of chronic conditions, body mass index, and study year.

**Result:**

Among the participants,. At baseline, Black men had a cognitive function score that was 1.14 points lower than White men (95% CI: ‐2.16, ‐0.12), after adjusting for sociodemographic and health factors. Over time, Black men experienced an additional decline of 0.12 points in cognitive function per year compared to White men (95% CI: ‐0.24, ‐0.001).

**Conclusion:**

The findings suggest that older non‐Hispanic Black men may experience accelerated cognitive aging at a faster rate compared to older White men. Policies and interventions promoting cognitive health should prioritize addressing these racial disparities in late adulthood.

